# Presence of the Extended-Spectrum-β-Lactamase and Plasmid-Mediated AmpC-Encoding Genes in *Escherichia coli* from Companion Animals—A Study from a University-Based Veterinary Hospital in Taipei, Taiwan

**DOI:** 10.3390/antibiotics10121536

**Published:** 2021-12-15

**Authors:** Fang-Ling Liu, Nan-Ling Kuan, Kuang-Sheng Yeh

**Affiliations:** 1 Graduate Institute of Veterinary Medicine, School of Veterinary Medicine, National Taiwan University, Taipei 10617, Taiwan; r05629015@ntu.edu.tw (F.-L.L.); nanikuan@gmail.com (N.-L.K.); 2 Biology Division, Animal Health Research Institute, Tansui, New Taipei City 25158, Taiwan

**Keywords:** extended-spectrum-β-lactamase, plasmid-mediated AmpC, *Escherichia coli*, companion animals

## Abstract

Extended-spectrum-β-lactamase (ESBL) and AmpC β-lactamase are two enzymes commonly found in *Enterobacteriaceae* that confer resistance to major antibiotics, such as third-generation cephalosporins that are widely prescribed for both human and animals. We screened for *Escherichia coli* producing ESBL and plasmid-mediated AmpC β-lactamase (pAmpC) from dogs and cats brought to National Taiwan University Veterinary Hospital, Taipei, Taiwan from 29 June 2020, to 31 December 2020. The genotypes and phylogenetic relatedness of these *E. coli* were also analyzed. Fifty samples of *E. coli* obtained from 249 bacterial isolates were included in this study. Among them, eight isolates had ESBL, seven had pAmpC, and one had both. Thirty-two percent (16/50) of *E. coli* isolates were resistant to third-generation cephalosporins. The detected ESBL genes included the *bla*_CTX-M-1_ and *bla*_CTX-M-9_ groups, and the *bla*_CMY-2_ group was the only gene type found in pAmpC. ESBL-producing *E. coli* belonged to the pathogenic phylogroup B2, and the sequence types (STs) were ST131 and ST1193. Three isolates were determined to be ST131-O25b, a highly virulent epidemic clone. The pAmpC-producing *E. coli* were distributed in multiple phylogroups, primarily the commensal phylogroup B1. The STs of the pAmpC-producing *E. coli* included ST155, ST315, ST617, ST457, ST767, ST372, and ST93; all of these have been reported in humans and animals. Imipenem was active against all the ESBL/pAmpC-producing *E. coli*; however, since in humans it is a last-resort antimicrobial, its use in companion animals should be restricted.

## 1. Introduction

Antimicrobial resistance (AMR) is a worldwide public health crisis that prevents practitioners, either from medicine or veterinary medicine, from successfully treating bacterial infections [1]. Members of the *Enterobacteriaceae* family, such as *Escherichia coli*, are responsible for most of the common infections in hospitals or communities [2]. β-lactamases are the most prominent enzymes in Gram-negative bacteria, and within the β-lactamase class, extended-spectrum-β-lactamases (ESBLs), AmpC (also termed class C) β-lactamases, and carbapenemases are currently widespread. These enzymes allow bacteria to resist the major therapeutic regimes available in clinical settings that rely on beta-lactams. Third-generation cephalosporins are broad-spectrum-β-lactam antimicrobials that are widely prescribed to both humans and animals to treat serious infections [3]. Because third-generation cephalosporins are essential treatments for many bacterial infections for which resistance is a major concern, this type of antimicrobial agents has been classified as “critically important” for human health [3].

Resistance to third-generation cephalosporins is often mediated by extended-spectrum-β-lactamase (ESBL) and AmpC β-lactamase. ESBLs are a group of enzymes derived from point mutations of β-lactamase. ESBLs confer resistance to most β-lactam antibiotics, including extended-spectrum cephalosporins and monobactams; ESBLs are, however, susceptible to carbapenems and cephamycins and are inhibited by clavulanic acid, sulbactam, and tazobactam [4]. AmpC β-lactamase is chromosomally mediated and present in various microorganisms [5]. The *ampC* gene in *E. coli* is normally expressed at a low level [6]. Papanicolaou et al. first demonstrated that AmpC β-lactamase was captured on a plasmid [7], and subsequently, plasmid-mediated AmpC β-lactamase (pAmpC) disseminated worldwide [5].

This study was conducted at National Taiwan University Veterinary Hospital (NTUVH), a teaching hospital affiliated with the College of Bioresources and Agriculture at National Taiwan University located in Taipei, Taiwan. The objective of this study was to screen for *E. coli* in the dogs and cats that visited NTUVH 29 June 2020, to 31 December 2020, examine their resistance to third-generation cephalosporins, and investigate the resistant gene profile of and connection among these isolates. The results provide valuable public health information.

## 2. Results

### 2.1. Occurrence of the ESBL/pAmpC-Producing E. coli and Their bla Genotypes

The 50 *E. coli* isolates obtained from this study (dog: *n* = 41, cat: *n* = 9) were initially screened using CHROMagar ESBL, after which the ESBL-producing *E. coli* were identified through the phenotypic confirmatory test. Nine *E. coli* isolates (dog: *n* = 6, cat: *n* = 3) that contained ESBL genes were discovered, one of which (from dogs) possessed both ESBL and pAmpC genes. Seven *E*. *coli* isolates contained only pAmpC genes. The *bla* genes that were identified included the *bla*_CTX-M-1_, *bla*_CTX-M-9_, and *bla*_TEM_ groups. The *bla*_CTX-M-1_ group contained *bla*_CTX-M-55_ (*n* = 2), *bla*_CTX-M-238_ (*n* = 2), *bla*_CTX-M-211_, and *bla*_CTX-M-199_. *bla*_CTX-M-235_ was the only type detected in the *bla*_CTX-M-9_ group. The *bla*_TEM_ group included *bla*_TEM-215_ (*n* = 4) and *bla*_TEM-243_ (*n* = 1). No *bla*_CTX-M-2_, *bla*_CTX-M-8_, *bla*_CTX-M-25_, or *bla*_SHV_ groups were found. Within the pAmpC gene groups, only the *bla*_CMY-171_ type of the *bla*_CMY-2_ group was detected (Table 1).

### 2.2. Phylogenetic Grouping, Genotyping, and Phylogenetic Analysis

**Figure 1:**
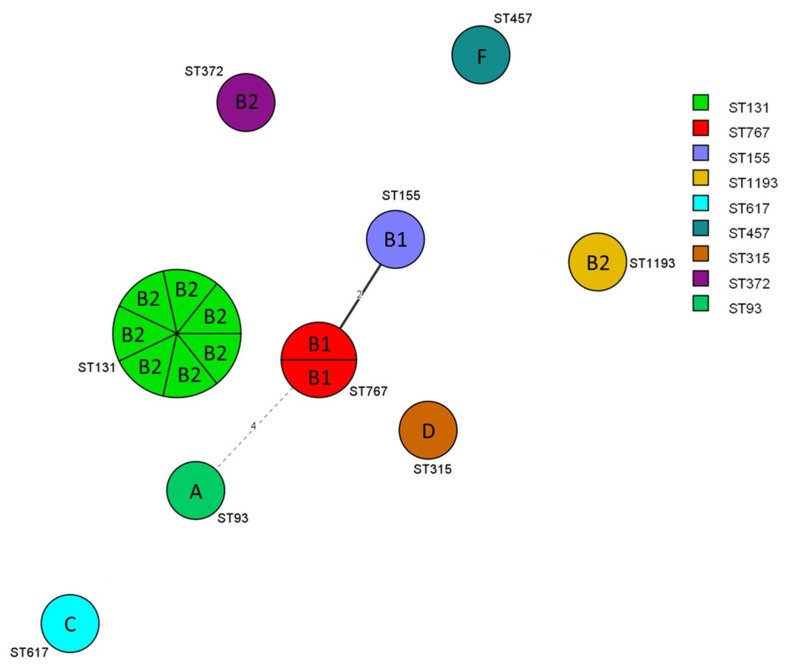
Minimal spanning tree of ESBL- and/or pAmpC-producing *E. coli*. Each circle indicates one sequence type (ST), divided into one sector for each isolate. The phylogenetic group is displayed within the sector, and each circle is bordered by the ST number. The numbers on the connecting line between STs within the MSTree indicate the number of different alleles. Solid and dotted lines represent allele differences of ≤3 and 4, respectively; allele differences >4 are not displayed.

B2 (*n* = 9) was the most common phylogroup, followed by B1 (*n* = 3). A, C, D, and F were detected once, and E and clade I were not detected. MLST identified nine STs among 16 ESBL- and/or pAmpC-producing *E. coli*, with ST131 being the most predominant ST (*n* = 7). ST767 was found in two *E. coli* isolates, and the other STs were encountered once (Table 1). Figure 1 demonstrates the minimal spanning tree of the 16 ESBL- and/or pAmpC-producing *E. coli* STs or phylogroups, according to the degree of allele sharing.

### 2.3. E. coli ST131 O25b Detection

Case numbers 004, 034, and 042 of the 7 ST131 ESBL-producing *E. coli* isolates tested positive for O25b clones, with both *trpA* and *pabB* PCR products observed on an agarose gel (Figure S1 in Supplementary Materials).

### 2.4. Conjugation Test

For the *E. coli* that only contained the pAmpC gene, a transfer of the *bla*_CMY-171_ gene to the recipient *E. coli* J53 strain was observed for five of six isolates by conjugation. The 049 strain did not transfer *bla*_CMY-171_ to the recipient strain. The 025 strain possessed both *bla*_TEM-243_ and *bla*_CMY-171_ and only transferred *bla*_CMY-171_ to the *E. coli* J53 strain. For the eight *E. coli* strains that possessed only ESBL genes, seven isolates could transfer all the ESBL genes to the *E. coli* J53 strain; however, only two of the three *bla* genes were transferred to the recipient *E. coli* by the 011 *E. coli* strain (Table 2). Figure S2 in Supplementary Materials demonstrates that the *bla* genes can be detected from the donor *E. coli* 031 strain and the transconjugant strain, but they cannot be detected in the recipient *E. coli* J53 strain.

### 2.5. Antimicrobial Susceptibility Test

**Figure 2:**
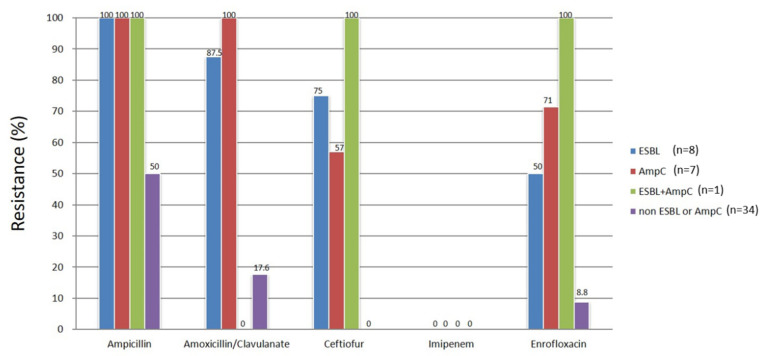
Antimicrobial susceptibility tests of *E. coli* containing ESBL, pAmpC, both, or neither. *E. coli* isolates that possess only ESBL, only pAmpC, both ESBL and pAmpC, and neither of the ESBL and pAmpC genes are represented by separate colors. The numbers adjacent to the end of the bars denote the percentage of resistance.

The results of the resistant rate of the 50 *E. coli* isolates to the specified antimicrobials are presented in Figure 2. The breakpoints and the details can be found in Supplementary Table (Tables S1 and S2). The *E. coli* isolates that carried the ESBL genes, pAmpC genes, or both all exhibited a more frequently multidrug-resistant phenotype than those that possessed neither gene. The *E. coli* isolates that possessed neither ESBL nor pAmpC were susceptible to ceftiofur and imipenem, and all of the *E. coli* isolates were susceptible to imipenem.

## 3. Discussion

Overall, 18% (9/50) of the *E. coli* assayed contained ESBLs, which was close to the percentage we previously reported (22.8%) [8]. We did not observe significant variations in the prevalence of ESBL-producing *E. coli* in companion animals in the same vicinity over time. Recently, Salgado-Caxito et al. reached the same conclusion through a scoping review and meta-analysis [9]. Although the prevalence did not fluctuate greatly over time, our data exhibited a higher rate than the average rate (6.87% in dogs and 5.04% in cats) reported from other continents, which could have resulted from our use of a dissimilar study design or methodology [9,10]. Additionally, NTUVH is a university-based teaching hospital and a major referral hospital for local clinics in Taipei. Therefore, previous antibiotic treatments prescribed to ailing dogs and cats before their admittance to NTUVH could also have contributed to a higher prevalence of ESBL-producing *E. coli*. In Asia, similar studies have revealed that the prevalence rates of ESBL-producing *E. coli* from companion animals in China and Japan were 24.5% and 28%, respectively [11,12]. In a study conducted in Pakistan, 15.3% of *Enterobacteriaceae* in companion animals were noted to be ESBL producing [13]. A high prevalence of ESBL-producing *E. coli* in Asian countries warrants concern. Socioeconomic and behavioral factors and veterinarians’ use of various levels of antibiotics may contribute to the disparate prevalence rates between Asia and other continents [9,14].

Eight isolates from 50 *E. coli* (16%) specimens contained pAmpC and belonged to different phylogroups (A, B1, B2, C, F, D), within which one isolate Case No. 002 possessed both pAmpC and ESBL. All the pAmpC genes of these eight isolates were determined to be *bla*_CMY-2_ group, which indicates that CMY-2 is the most prevalent and geographically diverse group of pAmpC enzymes [15,16,17]. Interestingly, our sequencing results revealed that *bla*_CMY-171_ was the only type within the *bla*_CMY-2_ group.

Thirty-two percent (16/50) of the *E. coli* assayed possessed ESBL- and/or pAmpC-encoding genes, a higher rate than those reported in Japan, Europe, or the United States [18,19,20,21,22]. The high frequency of ESBL and pAmpC genes in *E. coli* found in dogs and cats may pose a risk for the transmission this multidrug-resistant *E*. *coli* to pet owners. A study examined the genetic relationship between multidrug-resistant bacterial isolates, including ESBL producers, between pet owners and their dogs, demonstrating that 9.5% (4/42) of the owner–dog pairs shared similar multidrug-resistant *E. coli* isolates [23].

We only detected *bla*_CTX-M-1_, *bla*_CTX-M-9_, and *bla*_TEM_ groups from the ESBL-producing *E. coli*. Here, only *bla*_CTX-M-1_ and *bla*_CTX-M-9_ groups belonged to ESBL-encoding genes, whereas *bla*_TEM-215_ and *bla*_TEM-243_ were class A β-lactamase (https://www.ncbi.nlm.nih.gov/pathogens/beta-lactamase-data-resources/, accessed on 1 December 2021). *bla*_CTX-M-2_, *bla*_CTX-M-8_, *bla*_CTX-M-25_, and *bla*_SHV_ groups were not found; however, *bla*_CTX-M-2_ and *bla*_SHV_ groups had been present in the *E. coli* isolates obtained from the companion animals visiting NTUVH during studies conducted from 2014 to 2017 [8]. It is unknown why *E. coli*-containing *bla*_CTX-M-2_ and *bla*_SHV_ groups were absent during the present study. Teunis et al. conducted a longitudinal study to investigate the duration of the presence of ESBL- and pAmpC-producing *E. coli* in humans living in a livestock-dense region [24]. They concluded that *bla*_SHV-12_ was both easily acquired and lost. The estimated overall mean time to eliminate *bla*_SHV-12_ was 1.1 years, and the mean time to acquire it was approximately 3.0 years [24]. Because both this and our previous studies were only sectional studies, it is worth performing a longitudinal study in the future to investigate how long companion animals carry ESBL- or pAmpC-producing *E. coli*.

Of the nine ESBL-producing *E. coli* strains, seven were *E. coli* ST131 samples that were isolated from urine and ascites and belonged to the B2 phylogroup. Three out of the seven ST131 strains were identified as O25b clones, a globally spreading clone with a high virulence potential [25]. ST131 O25b with CTX-M-15 is a highly virulent clone for humans and is spreading globally [25]. The presence of this virulent clone was first reported in a dog with chronic cystitis in Portugal [26]. In our previous study, CTX-M-174 and CTX-M-194 were the β-lactamases found in the ST131 O25b clones [8], whereas CTX-235 and CTX-238 were those noted in the present study. β-lactamase other than CTX-M-15 were also found in *E. coli* ST131 O25b clones from companion animals [27]. Therefore, regardless of the β-lactamases present in the ST131 O25b clone, its potential zoonotic risk must be considered as a precaution. ST1193 has emerged as a pandemic clone of a multidrug-resistant human pathogen [28,29]. This ST was first identified in Australia as a fluoroquinolones resistant clone group [30], and its presence has been reported worldwide [31,32,33,34]. The 002 strain possessed both *bla*_CTX-M-55_ and *bla*_CMY-171_ and was typed as ST93, which has been reported as an avian and human extraintestinal pathogenic or diarrhoeagenic form of *E. coli* in humans and animals [35,36,37].

The conjugation test revealed that 81% (13/16) of the ESBL/pAmpC determinants were successfully transferred from the donor strains to the recipient *E. coli* J53 strain. The 049 strain did not transfer its single *bla*_CMY-171_ to *E. coli* J53, and the 011 and 025 strains transferred some *bla* genes to the recipient strain. The *bla* genes that did not transfer to the recipient strain may be located at plasmids other than those that transferred to the recipient strain. Studies have shown that some plasmids carrying *bla*_pAmpC_ were not self-transmissible; they can, however, be transferred through transformation or mobilization [5,38,39]. We cannot rule out the possibility that *bla*_CMY-171_ may be chromosome-encoded. Although plasmid extraction from the donor strains was performed (Figure S3), PCR-based replicon typing was not conducted which is a study limitation. Nevertheless, the conjugation test performed in this study demonstrated that the majority of *bla*_ESBL_- and *bla*_pAmpC_-encoding genes were located on mobile genetic elements, which has important public health implications because of its likely easier dissemination.

The ESBL/pAmpC-producing *E. coli* exhibited a more prominent multidrug-resistant phenotype than *E. coli* isolates without either gene. Imipenem was active against all *E. coli* strains in our study. This carbapenem class of drug has been used to treat multidrug- resistant bacteria in veterinary medicine [40]; however, it should be avoided and restricted to exceptional circumstances where no other options are available under the cascade because carbapenems are critically important antimicrobials of last resort for humans. Furthermore, carbapenem-resistant *E. coli* has been isolated from companion animals in previous studies, and thus, the selection pressure through antimicrobial use in companion animals should be avoided [41,42].

## 4. Materials and Methods

### 4.1. Sample Collection

Between June and December 2020, we cultured 249 bacterial isolates from 172 cases of companion animals admitted to NTUVH. Of them, 50 *E. coli* isolates were obtained from dogs (*n* = 41) or cats (*n* = 9). The bacteria were identified to the species level using a Vitek-2 Compact microbial detection system (bioMérieux, Marcy I’Etoile, France). The *E. coli* isolates were cultured from urine (*n* = 38), ascites (*n* = 2), pus (*n* = 4), nasal discharge (*n* = 2), body mass (*n* = 3), and an unknown source (*n* = 1). The isolates were stored in a Microbank system (Pro-Lab Diagnostics, Richmond Hill, ON, Canada) and maintained at −80 °C until analysis.

### 4.2. ESBL Screening and Phenotype Confirmation

*E. coli* obtained from NTUVH were streaked on CHROMagar ESBL plates (CHROMagar, Paris, France) to initially screen for ESBL producers. ESBL-producing *E. coli* would grow well and exhibit purple colonies on the chromogenic agar medium, and *E. coli* isolates that did not produce ESBL would not grow [43]. Subsequently, ESBL-producing *E. coli* were further identified by using the phenotypic confirmatory test specified by the Clinical and Laboratory Standards Institute (CLSI) [44]. *E. coli* that tested positive for ESBLs through the CHROMagar ESBL-test were evenly streaked on a Muller–Hinton agar (Difco/BectonDickinson, Franklin Lakes, NJ, USA) at a concentration of approximately 0.5 McFarland standards with a cotton swab. Four discs were placed on the agar surface: cefotaxime (30 μg), cefataxime–clavulanic acid (30 μg/10 μg), ceftazidime (30 μg), and ceftazidime–clavulanic acid (30 μg/10 μg). The plates were then incubated at 35 °C for 16 to 18 h. A difference of 5 mm or more in the inhibition zones between either cefotaxime–clavulanic or ceftazidime–clavulanic acid combination and cefotaxime or ceftazidime alone was used to identify an ESBL-producing *E*. *coli*. *Klebsiella pneumoniae* ATCC 700603 and *E. coli* ATCC 25922 were used as the positive and negative controls, respectively. The antimicrobial susceptible test discs were purchased from BD BBL (Difco/Becton Dickinson, Franklin Lakes, NJ, USA).

### 4.3. Phylogenetic Grouping

To understand the genetic substructure of the ESBL- and/or pAmpC-producing *E*. *coli*, the PCR method reported by Clermont et al. was applied to classify these *E. coli* cultures into multiple phylogroups, including A, B1, B2, C, D, E, F, and clade I [45]. The lysate preparations of the ESBL- and/or pAmpC-producing *E. coli* were used as the templates for the PCR, and the primers used are listed in Table 3. The boiling method reported by Shaheen et al. was used to prepare the lysates [46]. Briefly, the tested *E. coli* strains were cultured for 16–18 h at 37 °C on tryptic soy agar plates (Difco/Becton Dickinson, Franklin Lakes, NJ, USA). A loopful of bacterial cells was resuspended in 200 μL of double-distilled H_2_O (ddH_2_O) and boiled for 10 min. The supernatant was saved after centrifugation at 12,000× *g* for 10 min and used as the template source for PCR.

### 4.4. Genotyping and Phylogenetic Analysis

The ESBL- and/or pAmpC-producing *E. coli* were genotyped through multilocus sequence typing (MLST) [58]. The related PCR products were sequenced. The sequence data were then uploaded to the EnteroBase MLST website (http://enterobase.warwick.ac.uk/; accessed on 20 March 2021) for comparison. The similarities between these strains were analyzed using BioNumerics version 7.0 (Applied Maths, Sint-Martens-Latem, Belgium).

### 4.5. E. coli ST131 O25b Detection

*E. coli* ST131/O25b was detected through PCR based on the method described by Clermont et al. as follows: initial denaturation at 94 °C for 4 min followed by 30 cycles at 94 °C for 5 s, annealing at 65 °C for 10 s, and 72 °C extension for 5 min [52]. Ten μL of each PCR sample was inserted into a 2.0% agarose gel and electrophoresed at 100 V for 30 min using Tris-acetate-EDTA (TAE) buffer. The gels were then stained with a fluorescent nucleic acid dye (Biotium, Fremont, CA, USA) for 20 min and examined under ultraviolet illumination.

### 4.6. Conjugation Test

A conjugation test was performed using broth mating experiments as described by Tamang et al. [59]. We added 0.5 mL of the overnight culture of ESBL- and/or pAmpC-producing *E. coli* (donor) and *E. coli* J53 (recipient) to 4.5 mL of MH broth (Difco/Becton Dickinson, Franklin Lakes, NJ, USA) and incubated them with constant shaking at 37 °C for 4 h. An aliquot of 0.5 mL of the donor and recipient cells were added to 4 mL of MH broth and incubated with constant shaking at 37 °C overnight. An aliquot of 100 μL of the cocultured cells was spotted and evenly spread on the agar surface of the MH agar supplemented with sodium azide (150 mg/L) (Sigma) and cefotaxime (2 mg/L). Only transconjugants would grow on such double-selected MH agar, and neither the donor nor the recipient would be recovered. PCR detection for the specific ESBL and/or pAmpC genes of each donor strain was performed on the transconjugant strain to confirm the transfer of these genes.

### 4.7. Antimicrobial Susceptibility Test

All 50 *E. coli* isolates were tested for susceptibility to the specific antimicrobial agents using the Vitek 2 AST-GN96 card (bioMérieux, Marcy I’Etoile, France), which was designed for minimum inhibitory concentration (MIC) determination and for veterinary use only. We only included ampicillin, amoxicillin/clavulanate, ceftiofur, imipenem, and enrofloxacin in this study. Breakpoints specified in the Performance Standards for Antimicrobial Disk and Dilution Susceptibility Tests for Bacteria Isolated from Animals of CLSI (Vet 08) were used to interpret the data [44].

## 5. Conclusions

The expansion of the resistant genes mediated by plasmids, such as ESBL and pAmpC-encoding, has become a major public health concern. ESBL and pAmpC confer resistance to broad-spectrum cephalosporins, limiting treatment options in human and veterinary medicine. *E. coli* isolates of public health concern ST131 O25b, were discovered in this study, although they possessed *bla* genes other than *bla*_CTX-M-15_, which is commonly present in humans. ST131 O25b clones with different *bla* genes have been reported to be of companion animal origin. Thus, the role these animals may play in disseminating this clone should be considered. By frequently being MDR, the dissemination of such strains may lead to therapeutic failures or limited therapeutic options. A high prevalence of ESBL- and/or pAmpC-encoding genes in *E. coli* from companion animals underscores the necessity of antibiotic prudent use and periodic monitoring of multidrug-resistant bacteria.

## Figures and Tables

**Table 1:** *bla* genotypes, phylogroups, and ST types of ESBL/pAmpC-producing *E. coli* (*n* = 16).

Case No.	Species	Source	Phylogroup	ST Type	*bla* Genes
001	cat	urine	B2	ST131	*bla*_TEM-215_ + *bla*_CTX-M-235_ (ESBL)
002	dog	urine	A	ST93	*bla*_CTX-M-55_ + *bla*_CMY-171_ (ESBL + pAmpC)
004	cat	ascites	B2	ST131	*bla*_CTX-M-235_ (ESBL)
008	dog	urine	B1	ST155	*bla*_CMY-171_ (pAmpC)
010	dog	urine	D	ST315	*bla*_CMY-171_ (pAmpC)
011	cat	urine	B2	ST131	*bla*_TEM-215_ + *bla*_CTX-M-55_ + *bla*_CTX-M-235_ (ESBL)
025	dog	nasal discharge	B1	ST767	*bla*_TEM-243_ + *bla*_CMY-171_ (pAmpC)
031	dog	urine	B2	ST131	*bla*_TEM-215_ + *bla*_CTX-M-211_ + *bla*_CTX-M-235_ (ESBL)
032	cat	pus	C	ST617	*bla*_CMY-171_ (pAmpC)
034	dog	urine	B2	ST131	*bla*_CTX-M-238_ (ESBL)
038	dog	urine	F	ST457	*bla*_CMY-171_ (pAmpC)
040	dog	urine	B2	ST131	*bla*_CTX-M-235_ (ESBL)
042	dog	urine	B2	ST131	*bla*_CTX-M-238_ (ESBL)
049	dog	nasal discharge	B1	ST767	*bla*_CMY-171_ (pAmpC)
050	dog	oral mass	B2	ST1193	*bla*_TEM-215_ + *bla*_CTX-M-199_ (ESBL)
051	dog	urine	B2	ST372	*bla*_CMY-171_ (pAmpC)

**Table 2:** PCR detection of *bla* gene in donor and transconjugant strains in a conjugation test.

Case No.	*bla* Genes in the Donor Strain	*bla* Genes in the Transconjugant Strain
001	*bla*_TEM-215_, *bla*_CTX-M-235_	*bla*_TEM-215_, *bla*_CTX-M-235_
002	*bla*_CTX-M-55_, *bla*_CMY-171_	*bla*_CTX-M-__55_, *bla*_CMY-171_
004	*bla*_CTX-M-235_	*bla*_CTX-M-235_
008	*bla*_CMY-171_	*bla*_CMY-171_
010	*bla*_CMY-171_	*bla*_CMY-171_
011	*bla*_TEM-215_, *bla*_CTX-M-55_, *bla*_CTX-M-235_	*bla*_TEM-215_, *bla*_CTX-M-235_
025	*bla*_TEM-243_, *bla*_CMY-171_	*bla*_CMY-171_
031	*bla*_TEM-215_, *bla*_CTX-M-211_, *bla*_CTX-M-235_	*bla*_TEM-215_, *bla*_CTX-M-211_, *bla*_CTX-M-235_
032	*bla*_CMY-171_	*bla*_CMY-171_
034	*bla*_CTX-M-238_	*bla*_CTX-M-238_
038	*bla*_CMY-171_	*bla*_CMY-171_
040	*bla*_CTX-M-235_	*bla*_CTX-M-235_
042	*bla*_CTX-M-238_	*bla*_CTX-M-238_
049	*bla*_CMY-171_	-- ^†^
050	*bla*_TEM-215_, *bla*_CTX-M-199_	*bla*_TEM-215_, *bla*_CTX-M-199_
051	*bla*_CMY-171_	*bla*_CMY-171_

^†^: not detected.

**Table 3:** Sequences of primers used in this study.

PCR Target	Primer	Sequences (5′-3′)	Annealing Tm (°C)	Predicted PCR Size (bp)	Reference
*bla*_TEM_	TEM-F	TCGGGGAAATGTGCGCG	55	972	[47]
	TEM-R	TGCTTAATCATGAGGCACC			
*bla*_SHV_	SHV-F	GCCTTTATCGGCCCTCATCAA	54	819	[48]
	SHV-R	TCCCGCAGATAAATCACCACAATG			
*bla*_CTX-M-1-group_	CTX-M-1-F	CCCATGGTTAAAAAATCACTGC	54	942	[49]
	CTX-M-1-R	CAGCGCTTTTGCCGTCTAAG			
*bla*_CTX-M-2-group_	CTX-M-2-F	CGACGCTACCCCTGCTATT	52	552	[50]
	CTX-M-2-R	CCAGCGTCAGATTTTTCAGG			
*bla*_CTX-M-8-group_	CTX-M-8-F	TCGCGTTAAGCGGATGATGC	52	666	[50]
	CTX-M-8-R	AACCCACGATGTGGGTAGC			
*bla*_CTX-M-9-group_	CTX-M-9-F	ATGGTGACAAAGAGAGTGCAAC	55	876	[51]
	CTX-M-9-R	TTACAGCCCTTCGGCGATGATT			
*bla*_CTX-M-25-group_	CTX-M-25-F	GCACGATGACATTCGGG	52	327	[50]
	CTX-M-25-R	AACCCACGATGTGGGTAGC			
*bla*_pAmpC_	CIT-M-F	TGGCCAGAACTGACAGGCAAA	64	462	[52]
	CIT-M-R	TTTCTCCTGAACGTCGCTGGC			
*bla*_pAmpC_	MOX-M-F	GCTGCTCAAGGAGCACAGGAT	64	520	[52]
	MOX-M-R	CACATTGACATAGGTGTGGTGC			
*bla*_pAmpC_	DHA-M-F	AACTTTCACAGCTGTGCTGGGT	64	405	[52]
	DHA-M-R	CCGTACGCATACTGGCTTTGC			
*bla*_pAmpC_	CMY-F	ATGATGAAAAAATCGTTATGCT	64	1146	[52]
	CMY-R	TTATTGCAGCTTTTCAAGAATGCG			
*chuA*	chuA.1b	ATGGTACCGGACGAACCAAC	59	288	[45,53]
	chuA.2	TGCCGCCAGTACCAAAGACA			
*yjaA*	yjaA.1b	CAAACGTGAAGTGTCAGGAG	59	211	[45]
	yjaA.2b	AATGCGTTCCTCAACCTGTG			
TspE4.C2	TspE4C2.1b	CACTATTCGTAAGGTCATCC	59	152	[45]
	TspE4C2.2b	AGTTTATCGCTGCGGGTCGC			
*arpA*	AceK.f	AACGCTATTCGCCAGCTTGC	59	400	[45,54]
	ArpA1.r	TCTCCCCATACCGTACGCTA			
*arpA*	ArpAgpE.f	GATTCCATCTTGTCAAAATATGCC	57	301	[55]
	ArpAgpE.r	GAAAAGAAAAAGAATTCCCAAGAG			
*trpA*	trpAgpC.1	AGTTTTATGCCCAGTGCGAG	59	219	[55]
	trpAgpC.2	TCTGCGCCGGTCACGCCC			
*trpA*	trpBA.f	CGGCGATAAAGACATCTTCAC	59	489	[56]
	trpBA.r	GCAACGCGGCCTGGCGGAAG			
*pabB*	O25pabBspe.F	TCCAGCAGGTGCTGGATCGT	65	347	[57]
	O25pabBspe.R	GCGAAATTTTTCGCCGTACTGT			
*trpA*	trpA.F	GCTACGAATCTCTGTTTGCC	65	427	[57]
	trpA2.R	GCAACGCGGCCTGGCGGAAG			

## Data Availability

Data are contained within the article or the Supplementary Materials. Sequence data presented in this study are also available on request from the corresponding author.

## Supplementary Materials

The following are available online at https://www.mdpi.com/article/10.3390/antibiotics10121536/s1, Figure S1: PCR detection of *E. coli* ST131/O25b clone. Figure S2: PCR detection of the transfer of the ESBL genes from donor *E. coli* isolate 031 to recipient *E. coli* J53 strain in a conjugation test. Figure S3: The plasmid DNA isolation from the donor and the recipient cells. Table S1: Antimicrobial susceptibility test of the *E. coli* containing ESBL, pAmpC, both or neither. Table S2: Interpretive categories and breakpoints used in the present study.
