# Supplementary material for: Presence of the Extended-Spectrum-β-Lactamase and Plasmid-Mediated AmpC-Encoding Genes in Escherichia coli from Companion Animals—A Study from a University-Based Veterinary Hospital in Taipei, Taiwan
Source: Antibiotics (Basel). 2021 Dec 15;10(12):1536. doi: 10.3390/antibiotics10121536 (PMC8698527; doi:10.3390/antibiotics10121536)
Supplement: Supplementary file 1 [file antibiotics-10-01536-s001.zip › antibiotics-1444807-supplementary.pdf]

antibiotics

Article

Presence of the Extended-Spectrum-β-Lactamase and Plasmid-Mediated AmpC-Encoding Genes in *Escherichia coli* from Companion Animals—A Study from a University-Based Veterinary Hospital in Taipei, Taiwan

Fang-Ling Liu [1], Nan-Ling Kuan [1,2] and Kuang-Sheng Yeh [1,*]

[1] Graduate Institute of Veterinary Medicine, School of Veterinary Medicine, National Taiwan University, Taipei 10617, Taiwan; r05629015@ntu.edu.tw (F.-L.L.); nanikuan@gmail.com (N.-L.K.)
[2] Biology Division, Animal Health Research Institute, Tansui, New Taipei City 25158, Taiwan
* Correspondence: ksyeh@ntu.edu.tw; Tel.: +886-2-33661289

Supplementary Figures

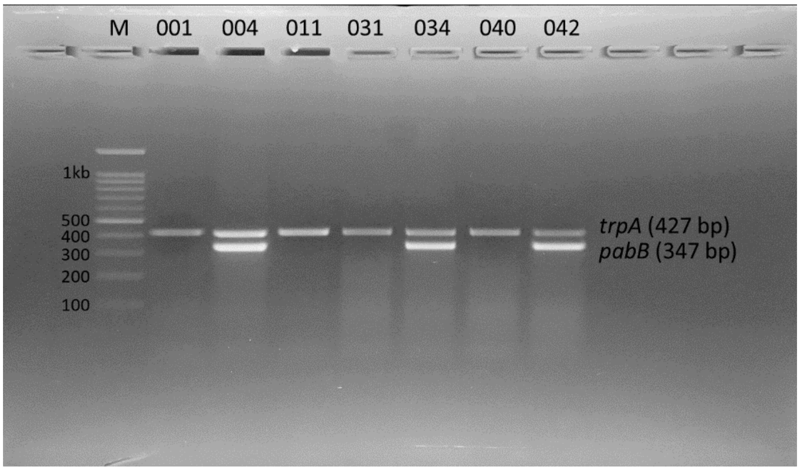

Figure S1. PCR detection of *E. coli* ST131/O25b clone. All ST131 isolates exhibit the *trpA* band with the molecular size of 427 bp, and the O25b clone possesses an additional 347 bp *pabB* band. Three ST131 isolates, 004, 034, and 042, were confirmed as O25b clones. M, molecular weight marker, 100 bp DNA ladder (Biomate, Taipei, Taiwan).

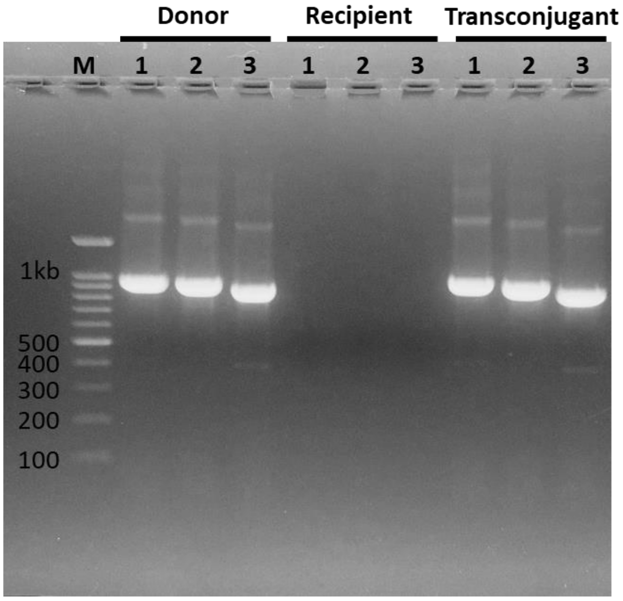

Figure S2. PCR detection of the transfer of the ESBL genes from donor *E. coli* isolate 031 to recipient *E. coli* J53 strain in a conjugation test. Cell lysates from the donor, recipient, and a transconjugant were prepared after conjugation and used as the templates in a PCR where the primers to amplify *bla*$_{TEM-215}$ (972bp), *bla*$_{CTX-M-211}$ (942bp), and *bla*$_{CTX-M-235}$ (876bp) were added. PCR products were detected in the donor *E. coli* isolate 031 and the transconjugant but not in the recipient *E. coli* J53 strain. M, molecular weight marker, 100 bp DNA ladder (Biomate, Taipei, Taiwan); 1, *bla*$_{TEM-215}$; 2, *bla*$_{CTX-M-211}$; 3, *bla*$_{CTX-M-235}$.

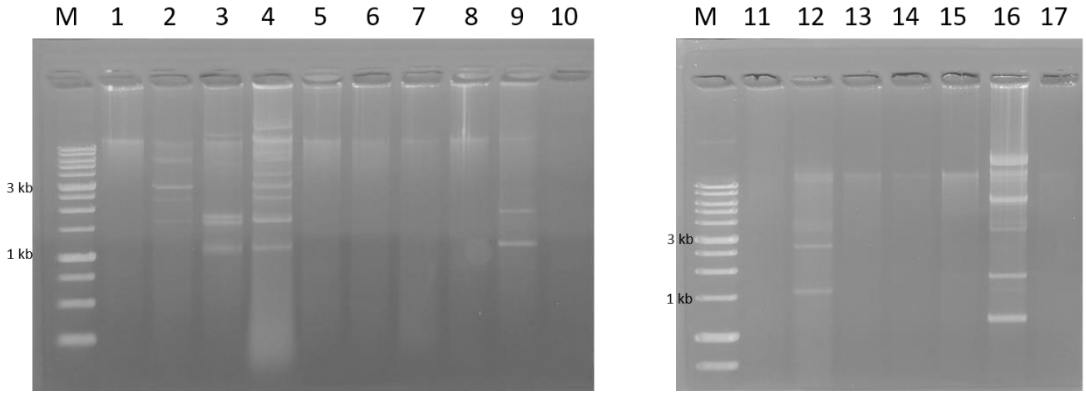

Figure S3. The plasmid DNA isolation from the donor and the recipient cells. Plasmid DNA preparations of the 16 ESBL-producing *E. coli* (donor) and *E. coli* J53 strain (recipient) were obtained using Monarch Plasmid Miniprep Kit (New England BioLabs, Ipswich, MA) according to the protocol provided by the manufacturer. Plasmid DNA was eluted in 100 µL of ddH_2O at the final step, and 10 µL of 3M sodium acetate and 1 mL of 95% ethanol were supplemented to precipitate DNA. The DNA preparation was stored at -80°C overnight, centrifuged for 10 min at 12,000 x g and the supernatant was removed. The DNA was washed with 70% ethanol and centrifuged for 10 min at 12,000 x g. After removing the supernatant, plasmid DNA was air dried and resuspended in 20 µL of ddH_2O. 10 µL of the plasmid preparation was electrophoresed onto an 1% agarose gel. The gel was stained with a fluorescent nucleic acid dye for 20 min and examined under UV light. M: 1 kb DNA ladder (Biomate, Taipei, Taiwan); 1, 001; 2, 002; 3, 004; 4, 008; 5, 010; 6, 011; 7, 025; 8, 031; 9, 032; 10, *E. coli* J53; 11, 034; 12, 038; 13, 040; 14, 042; 15, 049; 16, 050; 17, 051.

Supplementary Tables

Table S1. Antimicrobial susceptibility test of the *E. coli* containing ESBL, pAmpC, both or neither. **.**

**antimicrobials**	**ESBL (n=8)**			**pAmpC (n=7)**			**ESBL+pAmpC (n=1)**			**Non ESBL or pAmpC (n=34)**		
	R	I	S	R	I	S	R	I	S	R	I	S
ampicillin	8 (100)[a]	0 (0)	0 (0)	7 (100)	0 (0)	0 (0)	1 (100)	0 (0)	0 (0)	17 (50)	0 (0)	17 (50)
amoxicillin/clavulanate	7 (87.5)	1 (12.5)	0 (0)	7 (100)	0 (0)	0 (0)	0 (0)	0 (0)	1 (100)	6 (17.6)	5 (14.7)	23 (67.7)
ceftiofur	6 (75)	1 (12.5)	1 (12.5)	4 (57.1)	2 (28.6)	1 (14.3)	1 (100)	0 (0)	0 (0)	0 (0)	0 (0)	34 (100)
imipenem	0 (0)	0 (0)	8 (100)	0 (0)	0 (0)	7 (100)	0 (0)	0 (0)	1 (100)	0 (0)	0 (0)	34 (100)
enrofloxacin	4 (50)	2 (25)	2 (25)	5 (71.4)	0 (0)	2 (28.6)	1 (100)	0 (0)	0 (0)	3 (8.8)	1 (3.0)	30 (88.2)

R: resistant; I: intermediate; S: susceptible
a: the number in the parenthesis indicates the percentage.

Table S2. Interpretive categories and breakpoints used in the present study[a].

**antimicrobials**	**interpretive categories and MIC breakpoints, μg/mL**		
	**R**	**I**	**S**
ampicillin	≧1.0	0.5	≦0.25
amoxicillin/clavulanate	≧1.0	0.5	≦0.25
ceftiofur	≧8.0	4.0	≦2.0
imipenem[b]	≧4.0	2.0	≦1.0
enrofloxacin	≧4.0	1.0-2.0	≦0.5

[a]: The interpretive categories and breakpoints used: Vet 08, Performance Standards for Antimicrobial Disk and Dilution Susceptibility Tests for Bacteria Isolated from Animals, 4th ed. CLSI, 2020.
[b]: The breakpoints are for human. Imipenem is not licensed for veterinary use.
R: resistant; I: intermediate; S: susceptible.
